# LiF Reduces MICs of Antibiotics against Clinical Isolates of Gram-Positive and Gram-Negative Bacteria

**DOI:** 10.1155/2012/454065

**Published:** 2012-02-22

**Authors:** H. C. Syed, M. Ravaoarinoro

**Affiliations:** ^1^Faculté de Médecine Vétérinaire, Université de Montréal, Saint-Hyacinthe, QC, Canada; ^2^Département de Microbiologie-Immunologie, Université de Montréal, CRCHUM Hôpital Hôtel-Dieu de Montréal, 3840 rue St-Urbain, Montréal, QC, Canada H2W 1T8

## Abstract

Antibiotic resistance is an ever-growing problem yet the development of new antibiotics has slowed to a trickle, giving rise to the use of combination therapy to eradicate infections. The purpose of this study was to evaluate the combined inhibitory effect of lithium fluoride (LiF) and commonly used antimicrobials on the growth of the following bacteria: *Enterococcus faecalis*, *Staphyloccoccus aureus*, *Escherichia coli*, *Pseudomonas aeruginosa*, *Acinetobacter baumannii*, *Klebsiella pneumoniae*, *Serratia marcescens*, and *Streptococcus pneumoniae*. The *in vitro *activities of ceftazidime, sulfamethoxazole-trimethoprim, streptomycin, erythromycin, amoxicillin, and ciprofloxacin, doxycycline, alone or combined with LiF were performed by microdilution method. MICs were determined visually following 18–20 h of incubation at 37°C. We observed reduced MICs of antibiotics associated with LiF ranging from two-fold to sixteen-fold. The strongest decreases of MICs observed were for streptomycin and erythromycin associated with LiF against *Acinetobacter baumannii *and *Streptococcus pneumoniae*. An eight-fold reduction was recorded for streptomycin against *S. pneumoniae *whereas an eight-fold and a sixteen-fold reduction were obtained for erythromycin against *A. baumannii *and *S. pneumoniae*. This suggests that LiF exhibits a synergistic effect with a wide range of antibiotics and is indicative of its potential as an adjuvant in antibiotic therapy.

## 1. Introduction

The advent of penicillin, an antibiotic produced by the fungus *Penicillium,* marked a historic event in the medical field. Alexander Fleming first observed the antibiotic activity of penicillin against *Staphylococcus aureus* in Petri dishes. The discovery of penicillin, labeled the miracle drug, led physicians to believe the war with bacteria was over until the appearance of resistant strains. This led to the development of methicillin to combat infections caused by *S. aureus* strains resistant to penicillin. The cycle of creating new antibiotics and bacteria becoming resistant to them has contributed to the antibiotic resistance pandemic we are currently facing [1, 2]. The increase in antibiotic resistance coincided with the decline in the production of new antibiotics [3]. Accordingly, the use of combination therapy has evolved as an alternative to treat resistant pathogens. Combination therapy consists of using either two antibiotics or an antibiotic and an adjuvant to circumvent infections caused by pathogens resistant to antibiotics. Antibiotics used in combination therapy should act independently of each other. Successful combination therapy is characterized by a synergistic effect of the antibiotics or the antibiotic and the adjuvant demonstrated by a reduction in either the (MIC) minimal inhibitory concentration or (MBC) bactericidal concentration minimal of the antibiotic. In addition to reducing the dose of the antibiotic, combination therapy decreases the side effects of antibiotic therapy while slowing the emergence of resistance [3, 4].

The use of fluoride either alone or in combination therapy to combat caries has proved to be an effective practice in reducing the growth of cariogenic *Streptococci*. Fluoride inhibits enolase, a glycolytic enzyme coded by an essential gene and highly conserved among different forms of life ranging from archaebacteria and eubacteria to parasites and mammals [5, 6]. Enolase is a moonlighting protein which has been demonstrated to play roles in adhesion and in gene regulation [7, 8]. When used in combination therapy, fluoride is most commonly associated with cations which increase its antimicrobial properties. Different cations act as adjuvants to augment the antimicrobial activities of fluoride at varying degrees [9]. For example, AmF (amine fluoride) has been reported to be a stronger antimicrobial agent than NaF in the goal of eliminating caries-causing bacteria. The reason for this increased antimicrobial activity is not known but is thought to be related to a stronger inhibition of acid production by AmF [9, 10]. Furthermore, lithium exerts an adjuvant potential towards fluoride by increasing the antimicrobial properties of fluoride through poorly understood mechanisms [11]. Lithium has been employed as an adjuvant in oral vaccines against hepatitis B and acts by increasing the properties of aluminum in these vaccines [12].

The goal of this study was to determine the effects of LiF on antibiotics used against the planktonic form of the bacteria commonly associated with nosocomial infections: *Enterococcus faecalis*, *S. aureus*, *Escherichia coli*, *Pseudomonas aeruginosa*, *Acinetobacter baumannii*, *Klebsiella pneumoniae*, *Serratia marcescens,* and *Streptococcus pneumoniae* [2]. The antibiotics used in this study vary in the range of their target site and mode of action. We used members of the penicillin, *beta*-lactam, tetracycline, quinolone, macrolide, aminoglycoside, and sulfamide and trimethoprim families. Precisely, amoxicillin and ceftazidime were the penicillin and beta-lactam used, respectively, both of which inhibit peptidoglycan synthesis. Doxycycline was the tetracycline in our study while streptomycin and erythromycin were the aminoglycoside and macrolide, respectively; all three are protein synthesis inhibitors. Our quinolone, ciprofloxacin, interferes with DNA synthesis. Sulfamethoxazole, our sulfamide, was used with trimethoprim, to impede synthesis of folic acid [13]. We obtained reductions in MICs of antibiotics associated with LiF ranging from twofold to sixteenfold. The reductions in the MICs of all the antibiotics tested are indicative of the potential LiF has as an adjuvant in antibiotic therapy. Our sharpest reductions observed were with streptomycin and erythromycin against *S. pneumoniae*. The more pronounced reduction in the MICs of streptomycin and erythromycin associated with LiF against *S. pneumoniae* was the result of this organism being more susceptible to fluoride than the other bacteria used in this study.

## 2. Materials and Methods

### 2.1. Antimicrobial Agents

Standard antibiotic powders were purchased from Sigma-Aldrich Ltd (Montréal, Qc, Canada): ceftazidime, sulfamethoxazole-trimethoprim, streptomycin, erythromycin, amoxicillin, ciprofloxacin, and doxycycline. All of these compounds were diluted and stored according to the manufacturer's recommendations at −70°C until used.

### 2.2. Chemicals

Concentrated LiF (32 mg/L) stock solutions were prepared in double-deionized water, filter-sterilized, and stored in propylene containers. Stock solutions were diluted prior to use and were added to wells at a final concentration of 8 mg/L.

### 2.3. Test Organisms

The test organisms included eight American Type Culture Collection, (Rockville, MD, USA) ATCC strains which have been proposed as either quality control strains or reference strains: ATCC 29212 *Enterococcus faecalis*, ATCC29213 *Staphylococcus aureus*, ATCC49619 *Streptococcus pneumoniae*, ATCC 25922 *Escherichia coli*, and ATCC 27653 *Pseudomonas aeruginosa*, ATCC 19606 *Acinetobacter baumannii*, ATCC 13883 *Klebsiella pneumoniae*, ATCC 43861 *Serratia marcescens*.

### 2.4. Antibiotic Susceptibility Testing

Antibiotic susceptibility of our bacterial strains was tested by broth microdilution according to the guidelines from the Clinical and Laboratory Standards Institute [14]. Briefly, bacterial strains were grown in cation-adjusted Mueller-Hinton broth for 18–20 hours at 37°C. A bacterial suspension was prepared and adjusted to 0.5 McFarland turbidity standard (approximately 1 to 2 × 10^8^ CFU/mL). A 1 : 100 dilution followed by a 1 : 2 dilution was performed to yield a starting inoculum of 5 × 10^5^ CFU/mL. One hundred microliters of the bacterial suspension was added to each well of microdilution tray containing 50 *μ*L of LiF combined with 50 *μ*L of one of the above antimicrobials at a concentration equal to two times the final concentration. The trays were incubated at 37°C. MICs were recorded following 18–20 hours of incubation. The MIC resulted in complete visual inhibition of growth. Susceptibility tests were repeated on three separate occasions. Wells containing 200 *μ*L of fresh medium served as the negative control. The positive control consisted of wells inoculated with 100 microliters of the tested bacterial strain and 100 *μ*L of medium.

## 3. Results

The effect of LiF associated with antibiotics against eight ATCC strains of Gram-positive and Gram-negative bacteria is shown in Tables 1 and 2.

We observed reductions in MICs of antibiotics ranging from eightfold to twofold against the organisms tested above. An eightfold reduction in the MIC of DOX (doxycycline) against *S. aureus* and fourfold reductions of the MICs of CFT (ceftazidime) and TMX (sulfamethoxazole-trimethoprim) against *E. coli* were obtained. Twofold reductions in the MICs of DOX and TMX against *E. faecalis* were recorded as were twofold reductions in the MICs of CFT and TMX against *S. aureus* and *P. aeruginosa*. A twofold reduction in the MIC of DOX associated to LiF against *E. coli* was also noted (Table 1).

A sixteenfold reduction in the MIC of ERT (erythromycin) was obtained against *S. pneumoniae*. Eightfold reductions in the MICs of ERT against *A. baumannii* and of STP (streptomycin) against *S. pneumoniae* were observed. Fourfold reductions in the MICs of ERT and AMX (amoxicillin) against *K. pneumoniae* and in the MICs of AMX and CPF (ciprofloxacin) against *S. marcescens* and *S. pneumoniae* were noted. A greater-than-two-fold reduction in the MIC of AMX was observed against *A. baumannii*. Twofold reductions in the MICs of STP and CPF were observed against *A. baumannii *and* K. pneumoniae* as were twofold reductions in the MICs of STP and ERT against *S. marcescens* (Table 2).

## 4. Discussion

The goal of this study was to determine the adjuvant potential of LiF when associated with antibiotics commonly used in the clinical setting against bacteria frequently linked to nosocomial infections. The use of fluoride as an antimicrobial agent derives from the field of dentistry where it was first observed that fluoride arrested the growth of cariogenic streptococci by inhibiting the glycolytic enzyme enolase [6]. A study by Maehara et al. [15] demonstrated that fluoride and xylitol act synergistically to suppress sugar metabolism in *Mutans streptococci* leading to reduced growth of these organisms. We are pleased to report reductions in the MICs of all the antibiotics associated with LiF tested in our study. The association of LiF with doxycycline, ceftazidime, and sulfamethoxazole-trimethroprim against *E. faecalis*, *S. aureus*, *E. coli*, and *P. aeruginosa* was evaluated (Table 1). We also investigated the association with LiF to streptomycin, erythromycin, amoxicillin, and ciprofloxacin against *A. baumannii*, *K. pneumoniae*, *S. marcescens*, and *S. pneumoniae* (Table 2). We observed a twofold reduction in the MIC of doxycycline associated with LiF against *E. faecalis* and *E. coli* and a fourfold reduction against *S. aureus*. The greater decrease in the MIC of doxycycline suggests that this association is more effective in *S. aureus* than in *E. coli* and *E. faecalis*. We expected to see a greater reduction in the MIC against *E. coli* as enolase is a component of the mRNA degradosome in this organism and has been shown to bind the RNAse E within the degradosome [5]. Indeed, interactions between enolase and putative RNAses involved in mRNA degradation in *Bacillus subtilis* and *Streptococci pyogenes* have been reported suggesting a role in gene regulation for enolase [8, 16]. Although no interaction has been reported between the putative RNase involved in mRNA degradation and enolase in *S. aureus* [17], we nonetheless believe that an inhibition of enolase by LiF would interfere with gene regulation mechanisms in *S. aureus*. This is because a stronger reduction in the MIC of doxycycline associated with LiF against *S. aureus* was observed than those against *E. faecalis* and *E. coli*. Moreover, the twofold reductions in the MICs of ceftazidime and sulfamethoxazole-trimethoprim associated with LiF, neither of which targets protein synthesis and hence does not interfere with gene regulation mechanisms, against *S. aureus* suggest that the synergistic effect of LiF seems to be amplified in antibiotics interfering with gene regulation mechanisms.

We also examined the association of each ceftazidime and sulfamethoxazole-trimethoprim with LiF against *P. aeruginosa* and *E. coli.* A twofold reduction in the MIC of ceftazidime against *P. aeruginosa *was recorded while a fourfold reduction was obtained against *E. coli*. Hence, we were surprised to obtain a stronger reduction in the MIC of ceftazidime against *E. coli* than *P. aeruginosa* as ceftazidime is commonly used as an antipseudomonal agent [18]. The stronger reduction in the MIC of ceftazidime associated with LiF against *E. coli* suggests this organism is more strongly affected by an inhibition of enolase than *P. aeruginosa*, as demonstrated by our results. This could be explained by the fact that enolase is an abundant protein in *E. coli* compared to *P. aeruginosa*, a nonfermenter, which lacks glycolytic enzymes [19, 20]. Thus, *E. coli *would be more sensitive to an inhibition of glycolytic enzymes than *P. aeruginosa*. We also observed a twofold reduction in the MIC of sulfamethoxazole-trimethoprim associated with LiF against *E. faecalis* and *P. aeruginosa* and a fourfold reduction against *E. coli*. *E*. *coli* showed a greater susceptibility to the association of sulfamethoxazole-trimethoprim with LiF than *P. aeruginosa* and *E. faecalis*, with this phenomenon being reminiscent of the one observed for ceftazidime associated with LiF against *E. coli*. These results imply that inhibition of enolase increases the susceptibility of *E. coli* to antimicrobial agents possessing distinct mode of actions. The resolution of additional functions attributed to enolase would allow a better understanding as to how fluoride inhibition of enolase renders *E. coli* more susceptible to the action of antimicrobial agents. The reduction in the MICs of the antibiotics associated with LiF against *P. aeruginosa* indicates that LiF has targets other than enolase in this organism.

The reduction of the MICs of the streptomycin associated with LiF was twofold when used against *A. baumannii*, *K. pneumoniae*, and *S. marcescens* and eightfold against *S. pneumoniae* (Table 2). We believe the greater reduction of the MIC of streptomycin associated with LiF against *S. pneumoniae* could be caused by fluoride inhibiting multiple targets in this organism. Indeed, it has been demonstrated in *S. pyogenes* that fluoride downregulates the expression of the M-protein and glyceraldehyde 3-phosphate dehydrogenase (GAPDH) while suppressing the expression of the translation factor Tu [21]. We speculate that fluoride acts in a similar manner in *S. pneumoniae* as both *S. pneumoniae* and *S. pyogenes* belong to the same genus and, hence, are closely related. The inhibition of enolase and GAPDH as well as the suppression of Tu suggests that fluoride interferes with gene expression regulation mechanisms in this genus rendering them more susceptible to antibiotic treatment. In addition, the MIC of erythromycin associated with LiF against *S. pneumoniae* was reduced sixteenfold. The stronger reductions in MICs of streptomycin and erythromycin associated with LiF against *S. pneumoniae* are indicative of a greater susceptibility of this organism to this association. Champney et al. [22] have demonstrated that ketolides inhibit not only translation but also the assembly of the 50S ribosomal subunit in *S. pneumoniae*. The inhibition of both translation and the assembly of the 50S ribosomal subunit in *S. pneumoniae* by ketolides could explain the greater susceptibility to erythromycin of this organism compared to the other bacteria tested in our study. The reductions in the MICs of erythromycin associated with LiF were eightfold against *A. baumannii* and twofold against *K. pneumoniae* and *S. marcescens* (Table 2) implying that *A. baumannii*, like *S. pneumoniae*, is more susceptible to this association than *K. pneumoniae* and *S. marcescens*. Collectively, these results suggest streptomycin and erythromycin associated with LiF interfere with gene regulation mechanisms in a more pronounced manner in *S. pneumoniae* and *A. baumannii.*

The effect of LiF on amoxicillin against *A. baumannii*, *K. pneumoniae*, and *S. marcescens* was also verified. A greater-than two-fold reduction in the MIC of amoxicillin associated with LiF was observed against *A. baumannii*, followed by a fourfold reduction against *K. pneumoniae*, *S. marcescens*, and *S. pneumoniae* (Table 2). The greater reduction in the MIC of amoxicillin associated with LiF against *K. pneumoniae*, *S. marcescens*, and *S. pneumoniae* suggests that this association is more effective against these organisms than *A. baumannii*. The fourfold reduction obtained for *S. marcescens* and *K. pneumoniae* is reminiscent of the one observed against *E. coli* following a challenge of ceftazidime and trimethoprim-sulfamethoxazole each associated with LiF (Table 1). It is interesting to note that both ceftazidime and amoxicillin target cell wall synthesis and that when associated with LiF provoke a fourfold reduction against the *Enterobacteria*. These preliminary results suggest the association of LiF with antibiotics inhibiting cell wall synthesis could be effective against *Enterobacteria*.

The last association we tested was that of LiF with ciprofloxacin where we obtained a two-fold reduction in the MIC of ciprofloxacin against *A. baumannii* and *K. pneumoniae* and a fourfold reduction against *S. marcescens* and *S. pneumoniae* (Table 2). It is unclear why we observed a twofold reduction in the MIC of ciprofloxacin in combination with LiF against *K. pneumoniae* and a fourfold reduction against *S. marcescens*, as both of these organisms demonstrated an identical reduction in the MIC of amoxicillin associated with LiF. Further testing of the combination of ciprofloxacin and LiF against other *Enterobacteria* would be required to observe the effects of this association on these types of organisms. Moreover, ciprofloxacin is the only antibiotic used in this study targeting DNA synthesis and thus renders interpretation of the results difficult.

This preliminary study sheds light on the potential of LiF as an adjuvant in antibiotic therapy. Antibiotics targeting DNA, membrane, and protein synthesis all showed at least twofold reductions in their MICs against both Gram-negative and Gram-positive bacteria. Our results demonstrate that LiF could be used as a novel adjuvant in combination therapy with antibiotics to treat infections, particularly those caused by antibiotic-resistant microorganisms which normally require high doses of antibiotics, thus supporting the data obtained previously. Data from our laboratory have demonstrated that LiF alone inhibits bacterial growth by 30% in pseudomonal strains suggesting it possesses bacteriostatic activity (unpublished). This same study also revealed twofold reductions in the MICs of ceftazidime in combination with LiF and was used against pseudomonal strains, implying that LiF and ceftazidime act in synergy. Taken together, these results indicate that the association between ceftazidime and LiF represents a form of combination therapy against pseudomonal strains worth exploring. The study described here establishes a pronounced synergistic effect between LiF and erythromycin compared to the other antibiotics tested against *S. pneumoniae* and *A. baumannii*. Although these *in vitro* results are exciting, the effects of using combination therapy of LiF and antibiotics *in vivo* remain unknown. Furthermore, our results need confirmation by testing antibiotics other than the ones used here against the bacteria used in our study. For example, testing antibiotics targeting protein synthesis other than doxycycline in combination with LiF against *S. aureus, E. faecalis, *and* E. coli* would be required to confirm the idea that *S. aureus* is more susceptible to this type of association than are *E. faecalis* and *E. coli*. Likewise, testing the association of other antibiotics targeting the cell wall with LiF as well as the association of antibiotics targeting DNA synthesis in combination with LiF against *Enterobacteria* will allow a better understanding of the consequences of these associations on these bacteria. The association of antibiotics targeting the 50S ribosomal subunit with LiF against *A. baumannii*, *S. pneumoniae*, and other bacteria needs to be verified in order to determine if the synergistic effect of erythromycin and LiF demonstrated in our study is specific to this antibiotic. In spite of the limitations of our study, we nonetheless believe that the results obtained here hold the potential for the development of future combination therapies used to eradicate infections caused by drug-resistant bacteria. The development of combination therapy represents an alternative to prescribing high doses of antibiotics used to treat infections resulting from resistant pathogens.

## Figures and Tables

**Table 1:** MICs (*μ*g/mL) of doxycycline, ceftazidime, and sulfamethoxazole-trimethoprim alone or in combination with LiF on *Enterococcus faecalis*, *Staphylococcus aureus*, *Escherichia coli*, and *Pseudomonas aeruginosa*.

	Antimicrobial agents					
MIC (*μ*g/mL)	DOX	DOX + LiF	CFT	CFT + LiF	TMX	TMX + LiF
Bacterial strain						
*E. faecalis *ATCC 29212	8.0	4.0	NT	NT	0.125	0.06
*S. aureus *ATCC 29213	0.125	0.03	2.0	1.0	0.50	0.25
*E. coli *ATCC 25922	1.0	0.5	0.25	0.06	0.25	0.06
*P. aeruginosa *ATCC 27653	NT	NT	2.0	1.0	16.0	8.0

DOX: doxycycline, CFT: ceftazidime, TMX: sulfamethoxazole-trimethoprim, NT: not tested.

**Table 2:** MICs (*μ*g/mL) of streptomycin, erythromycin, amoxicillin, and ciprofloxacin alone or in combination with LiF on planktonic *Acinetobacter baumannii*, *Klebsiella pneumoniae*, *Serratia marcescens*, and *Streptococcus pneumoniae*.

	Antimicrobial agents							
MIC (*μ*g/mL)	STP	STP + LiF	ERT	ERT + LiF	AMX	AMX + LiF	CPF	CPF + LiF
Bacterial strain								
*A. baumannii *	128	64	128	16	>128	64	1	0.5
ATCC 19606								
*K. pneumoniae*	1.0	0.5	64	16	16	4	0.125	0.0625
ATCC 13883								
*S. marcescens*	16	8	16	8	8	2	0.25	0.0625
ATCC 43861								
*S. pneumoniae *	0.25	0.03	0.25	0.015	0.06	0.015	0.06	0.015
ATCC 49619								

STP: streptomycin, ERT: erythromycin, AMX: amoxicillin, CPF: ciprofloxacin.
